# VNTR Polymorphisms in the *SLC6A3* Gene and Their Impact on Time Perception and EEG Activity

**DOI:** 10.3390/bioengineering12101118

**Published:** 2025-10-19

**Authors:** Francisco Victor Costa Marinho, Silmar Silva Teixeira, Giovanny Rebouças Pinto, Thomaz de Oliveira, France Keiko Nascimento Yoshioka, Hygor Fernandes, Aline Miranda, Bruna Brandão Velasques, Alair Pedro Ribeiro de Souza e Silva, Maurício Cagy, Victor Hugo do Vale Bastos

**Affiliations:** 1 Brain Mapping and Functionality Laboratory, Federal University of Delta do Parnaíba, Parnaíba 64202-020, PI, Brazil; silmarteixeira@ufpi.edu.br (S.S.T.); aliness.miranda88@gmail.com (A.M.); victorhugobastos@ufdpar.edu.br (V.H.d.V.B.); 2 Neuro-Innovation Technology & Brain Mapping Laboratory, Federal University of Delta do Parnaíba, Parnaíba 64202-020, PI, Brazil; thomaz159@gmail.com; 3 Genetics and Molecular Biology Laboratory, Federal University of Delta do Parnaíba, Parnaíba 64202-020, PI, Brazil; pintogr@ufpi.edu.br (G.R.P.); keiko@ufdpar.edu.br (F.K.N.Y.); hygorf2@gmail.com (H.F.); 4 Brain Mapping and Sensory Motor Integration Laboratory, Institute of Psychiatry, Federal University of Rio de Janeiro, Rio de Janeiro 22290-140, RJ, Brazil; bruna_velasques@yahoo.com.br (B.B.V.); ribeiropss@yahoo.com.br (A.P.R.d.S.e.S.); mauricio.cagy@gmail.com (M.C.)

**Keywords:** time perception, time estimation, *SLC6A3* gene, dopamine, EEG alpha power

## Abstract

**Aim:** The research examined the relationship between *SLC6A3* 3′-UTR and intron 8 VNTR polymorphisms and their influence on supra-second time estimation performance and EEG alpha band activity. **Material and methods**: A total of 178 male participants (aged 18 to 32 years) underwent genotyping for the *SLC6A3* 3′-UTR and intron 8 VNTR polymorphisms. An electroencephalographic assessment was conducted targeting the dorsolateral prefrontal cortex (DLPFC), simultaneously with the time estimation task. The 3′-UTR and intron 8 VNTRs polymorphisms were linked to absolute error and ratio in a time estimation task (target duration: 1 s, 4 s, 7 s, and 9 s) neurophysiological variable. **Results**: Regarding the absolute error and ratio, the combinatory effect of *SLC6A3* 3′-UTR and intron 8 VNTRs showed a difference in the interpretation of time only for 1 s (*p* = 0.0002). In the EEG alpha power, the analysis revealed a difference only for the left DLPFC (*p* = 0.0002). **Conclusions**: Electrophysiological and behavioral investigation in the time perception associated with the *SLC6A3* gene suggests an alternative evaluation of neurobiological aspects inbuilt in timing. The 3′-UTR and intron 8 VNTR polymorphisms modulate dopaminergic neurotransmission during short-temporal scale judgment in the domain of supra seconds and indicate a role in its inputs to the left dorsolateral prefrontal cortex during the voluntary attention processes for visual stimulus. Our findings demonstrate that cognitive resources to capture and store time intervals can be measured based on the EEG power activity pattern.

## 1. Introduction

Human time estimation emerges from dynamic central nervous system (CNS) responses that encode and interpret environmental stimuli [1,2,3]. Therefore, temporal information encoding engages core cognitive mechanisms and multisensory integration processes [4,5], which require a complex mechanism of cortical oscillations during timing [6,7,8]. It is crucial to address dopaminergic neurotransmission underlying the time estimation in the domains of milliseconds-by-hours and days [9,10]. Dopaminergic activity modulates the interindividual internal clock [11,12]. It triggers neurofunctional responses in various aspects of time synchronism, which was suggested by studies involving pharmacological paradigms, brain mapping, and molecular biology [13,14,15,16,17].

Regarding the molecular influence during timing, tests with fixed-time intervals in knockout rodents for the gene *Solute carrier family 6 member 3* (*SLC6A3* gene) demonstrated less accuracy in the temporal judgment and changes in homeostatic activities (i.e., sleep cycle, perception of stimuli) due to the increase in extracellular dopamine levels [18,19]. Therefore, changes in the transcriptional control of dopaminergic pathways provide a foundation for understanding how the brain encodes durations exceeding one second at both neurophysiological and behavioral levels.

Within this framework, the *SLC6A3* 3′-UTR VNTR polymorphism (rs28363170), situated in exon 15 of the 3′ untranslated region, consists of 40-base pair tandem repeats ranging from 3 (3R) to 13 (13R), which modulate the functional expression of the dopamine transporter (DAT) in brain regions critical for temporal processing [20]. Among these variants, the 9-repeat (9R) and 10-repeat (10R) alleles are the most prevalent in the general population [21]. Individuals carrying the 9R variant of the DAT gene demonstrate reduced expression of the dopamine transporter protein in the dorsolateral prefrontal cortex (DLPFC) compared to those homozygous for the 10R allele [22]. Another widely studied variation, *SLC6A3* intron 8 VNTR (rs3836790), has 5 or 6 repeats of 30 bp (5R and 6R, respectively), whose 6R repeat is associated with transport deficiency and protein synthesis in dopaminergic reuptake, concerning 5R [20,23]. Accordingly, findings in behavioral genetics indicate that molecular information governing critical alterations in cognitive-related protein expression can modulate phenotypic traits involved in the processing of temporal intervals [24].

Investigations of the influence of *SLC6A3* 3′-UTR and intron 8 VNTR polymorphisms in timing studies are essential to unveil endophenotypes [24,25,26]. Numerous investigations have sought to identify genetic associations underlying neurophysiological mechanisms and behavioral outcomes by employing timing tasks involving visual and auditory stimuli across both human and non-human species [12,26,27,28,29]. In humans, variations in visual stimulus processing have been linked to prefrontal cortical dynamics, as reflected by electroencephalography (EEG)-derived measurements of alpha-band spectral power [30,31]. For instance, research conducted by Laakso et al. [32], Bender et al. [33], and Katz et al. [25] provided evidence that the EEG power of the EEG in frontal electrodes varies based on different genotypes that encode the level of DAT and serotonin transporter during spatial and cognitive processing based on associated phenotypic traits in the stimulus timing process. Nonetheless, the neurobiological mechanisms underlying temporal interval judgment from a genetic perspective remain insufficiently characterized. To date, no investigation has explored the combined influence of *SLC6A3* 3′-UTR and intron 8 VNTR polymorphisms on timing behavior and associated neurophysiological parameters.

Oscillatory neural dynamics represent cyclical fluctuations in neuronal responsiveness, spanning multiple spatial domains and temporal frequencies [34,35]. The investigation of electrophysiological changes related to time intervals’ interpretation is commonly established through the study of the alpha band power in the prefrontal cortex [36]. It allows us to relate whether the increase or decrease in the absolute power in this area was due to the changes in the attentional level and perception of visual stimuli directed to the task [36,37,38,39].

Therefore, assessing alpha-band power in the dorsolateral prefrontal cortex during temporal estimation tasks may serve as a neurophysiological marker of *SLC6A3* genetic variation and its influence on behavioral profiles related to supra-second time processing. This study hypothesizes that the interaction between the *SLC6A3* 3′-UTR and intron 8 VNTR polymorphisms may lead to variations in stimulus sensitivity, and, potentially resulting in distinct cognitive encoding of temporal intervals and corresponding modulations in EEG alpha-band power. Thus, this study attempts to analyze the combinatorial effect of *SLC6A3* 3′-UTR and intron 8 VNTRs genetic polymorphisms on the time estimation task and EEG alpha-band power in the DLPFC during perceptual capacity.

## 2. Materials and Methods

### 2.1. Participants

A total of 178 healthy male participants from a northeastern Brazilian population were recruited, all of whom were right-handed as confirmed by the Edinburgh Handedness Inventory [40], aged between 18 and 32 years (mean ± SD = 22.05 ± 3.07 years). Participants abstained from consuming any substances capable of affecting neural activity—such as tobacco, alcohol, caffeinated products, or pharmacological agents—for at least 14 h prior to and throughout the experimental procedures and were evaluated by the Mini-Mental State Examination (MMSE) (28 ± 0.9) and Cronbach’s alpha (α) = 0.79; *p* = 0.001. All participants were clinically screened to rule out neurological or motor disorders that could compromise task execution or interfere with accurate time perception.

The Ethics Committee of the UFPI approved all procedures, and participants were provided written, informed consent (#:1.087.450).

### 2.2. Genotyping

Genotyping procedures were conducted using a protocol adapted from Marinho et al. [17], which employed polymerase chain reaction (PCR) to detect the *SLC6A3* 3′-UTR and intron 8 VNTR polymorphisms.

Briefly, genomic DNA was extracted from peripheral blood leukocytes using the Wizard^®^ Genomic DNA Purification Kit (Promega, Madison, WI, USA), following the manufacturer’s instructions.

The analysis of *SLC6A3* 3′-UTR VNTR of 40 bp was performed using the polymerase chain reaction (PCR) using the *primers forward* 5′-TGT GGT GTA GGG AAC GGC GTG AG-3′ and *reverse* 5′-CCT CCT GGA GGT CAC GCG TCA AGG-3′. For a total reaction volume of 25 μL, the following conditions were used: 1.0 μL DNA, 2.5 μL of buffer 10x (50 mM KCl, 20 mM Tris-HCl/pH 8.4), 0.75 µL of MgCl_2_, 1.0 μL of each primer, 5.0 μL of dNTPs, 0.3 μL of Taq DNA polymerase, and 13.45 μL of distilled H_2_O was added to make up the final volume. The *SLC6A3* intron 8 VNTR of 30 bp was performed utilizing PCR using *primers forward* 5′-CCC AGG GAC ATC TGC TAA TG-3′and *reverse* 5′-CAC AAA TGA GTG TTC GTG CAT G-3′. For a total reaction volume of 25 μL, the following conditions were used: 1.0 μL DNA, 2.5 μL buffer 10× (50 mM de KCl, 20 mM Tris-HCl/pH 8.4), 0.9 µL de MgCl_2_, 0.4 µL of each primer, 4.0 μL of dNTPs, 0.2 μL of Taq DNA polymerase, and 15.6 µL of distilled H_2_O was added to make up the final volume. Primers were commercially manufactured by Thermo Fisher Scientific Inc. (Waltham, MA, USA), and all other reagents used were supplied by Ludwig Biotechnology Ltd. (Porto Alegre, RS, Brazil).

**Figure 1:**
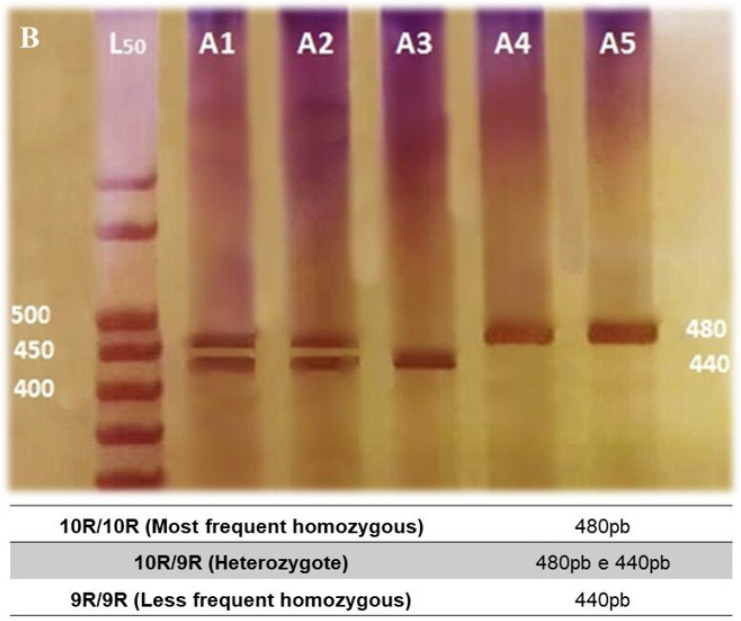
Representative genotyping of the *SLC6A3* 3′-UTR VNTR polymorphism using 8% polyacrylamide gel electrophoresis. Bands correspond to the 10R (480 bp) and 9R (440 bp) alleles. Negative control and molecular weight marker included. Note: B—White, L50—50 base pair ladder.

**Figure 2:**
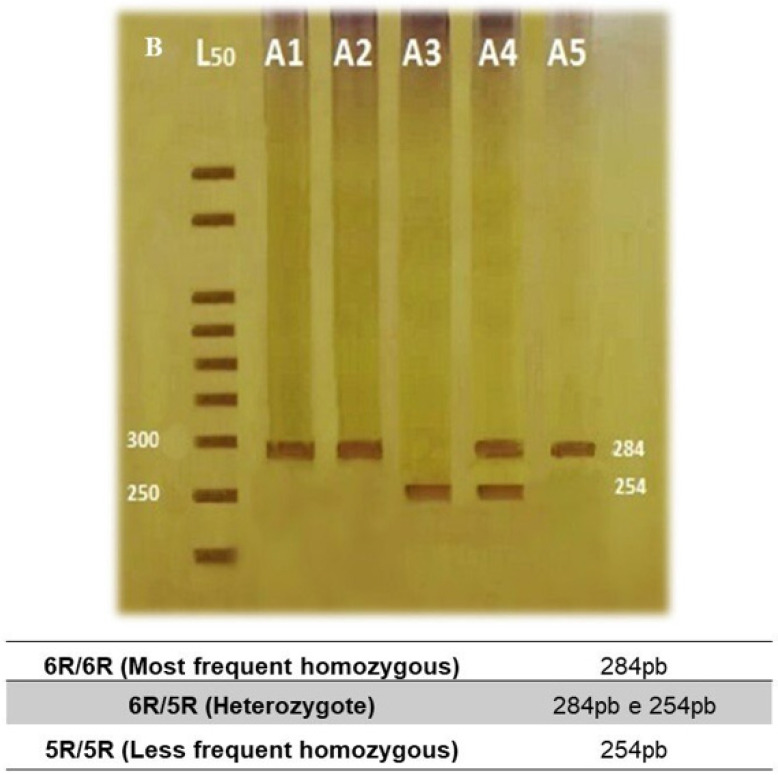
Representative genotyping of the *SLC6A3* intron 8 VNTR polymorphism. PCR products show the 6R (284 bp) and 5R (254 bp) alleles on 8% polyacrylamide gel electrophoresis. Negative control and molecular weight marker included. Note: B—White, L50—50 base pair ladder.

PCR was carried out as follows: initial denaturation at 95 °C for 5 min followed by 35 cycles at 95 °C for 45 s, 62 °C (3′-UTR) or 63 °C (intron 8) for 45 s, followed by 72 °C for 45 s, and a final extension at 72 °C for 7 min. The resultant PCR products for 10R and 9R alleles (479 bp and 439 bp) of *SLC6A3* 3′-UTR VNTR (Figure 1), and 6R and 5R alleles (254 bp and 284 bp) of intron 8 VNTR (Figure 2) were separated on an 8.0% polyacrylamide gels and visualized using silver nitrate staining. PCR quality control was performed by randomly selecting 20% of the samples for re-genotyping by an independent technician. The correlation observed for the genotyping assays was 100%.

### 2.3. Time Perception Task

**Figure 3:**
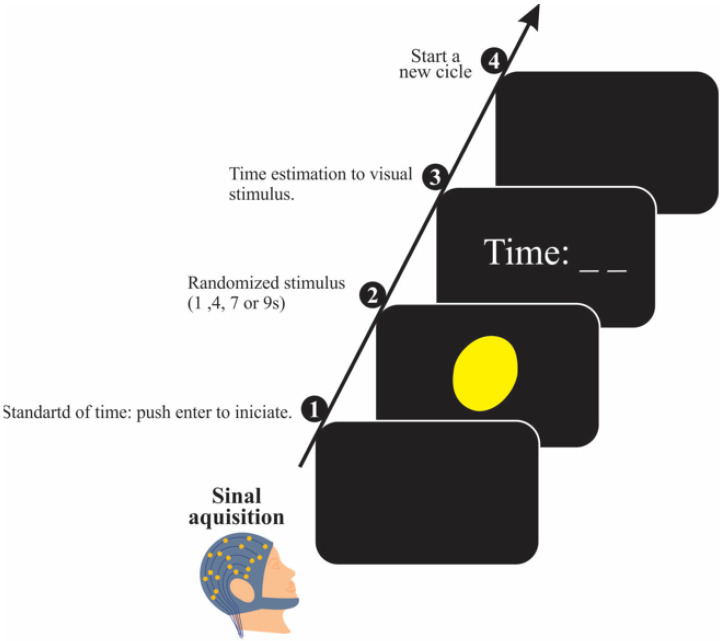
Experimental procedure of the time estimation task.

A 42-inch monitor was positioned on a Table 50 cm from the participant, remaining off except during task execution. Temporal estimation was assessed using a software application that registered the duration of visual stimuli presented at predefined intervals (e.g., 1 s, 4 s, 7 s, or 9 s) [17,39,41]. The task was performed in two phases. To begin, the display shows the command “enter”, then, the program produces a yellow circle in the monitor center that remains randomly for 1 s, 4 s, 7 s, or 9 s. Subsequently, the Matlab—R2020b software presented an empty input field on the screen, prompting the participant to manually enter their estimated duration before confirming the response by pressing the “enter” key (Figure 3). Each participant performed one block with 40 trials.

### 2.4. EEG Recording

**Figure 4:**
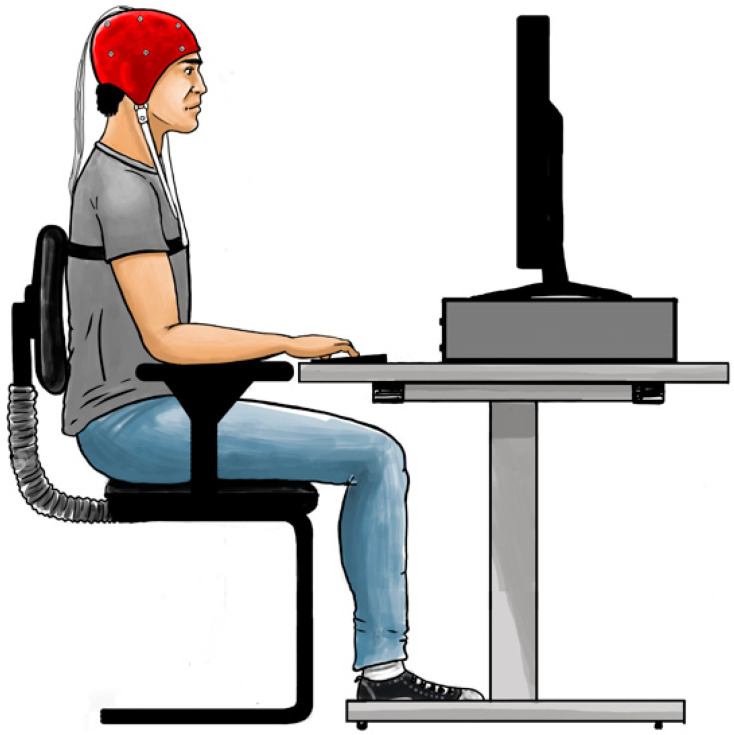
Subjects’ position during time estimation task execution and EEG recording.

All participants were positioned in a controlled environment featuring acoustic insulation, electrical shielding, and dim lighting. They remained seated in armrest-equipped chairs to reduce muscle-related artifacts during EEG data collection (Figure 4). The 20-channel continuous EEG was recorded by BrainNet BNT36 (EMSA Medical Equipment, EMSA, Lisbon, Portugal). The silver/silver chloride electrodes were positioned through a nylon cap following the international 10–20 system, including binaural reference electrodes (SPES Medical Brazil, Belo Horizonte, Brazil). The impedance of EEG and electrooculogram (EOG) electrodes were kept below 5 kΩ. The acquired data had an amplitude below 100 μV. The sampling rate was 240 Hz. An anti-aliasing low-pass filter with a cut-off frequency of 100 Hz was employed. It was configured to use 60 Hz Notch digital filtering, with high-pass filters at 0.03 Hz and low-pass filters at 40 Hz (Order 2 Butterworth filter), using the Data Acquisition software (Delphi 5.0) developed at the Neuro-innovation Technology (Maastricht, The Netherlands) and Brain Mapping Laboratory (Manhasset, NY, USA).

### 2.5. Behavioral Data

The behavioral variable was transformed into measures representing the absolute error value (AE) and the estimated proportion of target duration (Ratio) [42]. The AE value measures the difference between the target-time and judged time-intervals, making it useful to assess the timing precision. AE was calculated as the absolute value of the difference between the time estimation (Te) and the target duration (Td) [AE = |Te − Td|] [43]. The ratio was obtained by dividing each participant’s time performance by the time duration of the interval presented for that trial [Target duration estimation = Te/Td]. A coefficient below 1.0 indicates a trial of the time estimates less than the real-time; on the other hand, a coefficient above 1.0 indicates the time estimate to be longer than the real duration, an underestimation and overestimation of time, respectively [29,39].

The analysis of the coefficient of variation (CV) represents the variability in temporal judgments and shows the consistency of temporal performance and precision for the same target duration [16]. The CV was calculated by dividing the standard deviation (S) by the average time judgment for each time interval (|µ|) times one hundred: CV = S/|µ| × 100. In this study, the variability statistic indicates that the lower the estimate, the greater the test’s precision, while the higher the estimate, the lower the precision. The CV classification was considered short CV when less than 10%; medium, in the range of 10 and 20%; high, between 20 and 30%; and very high CV when they are above 30% [44].

### 2.6. Genetic Data

The analysis was guided by the functional impact of the polymorphisms on protein expression levels [21,33]:

(a) Combinatory effect between *SLC6A3* 3′-UTR and intron 8 VNTRs for DAT signaling (*n* = 174): Cluster 10R-10R/6R-6R (*n* = 71) vs. Grouping no 10R-10R/6R-6R (*n* = 103) [21,22,33]. The other genotypes of the 3′-UTR and the intron 8 VNTRs (four samples in present study) considered without functionality in dopaminergic regulation were excluded [33,45].

### 2.7. EEG Data Processing

EEG power analysis is based on a direct proportionality in which higher signal amplitude reflects greater spectral power. Absolute power, quantified in microvolts (μV), represents the energy magnitude within a specific frequency band recorded at designated electrode sites [46].

Artifact removal was performed using a hybrid approach that integrated manual visual inspection with independent component analysis (ICA) using Matlab 5.3^®^ (The MathWorks, Inc., Carlsbad, CA, USA). Signals from electrodes exhibiting poor scalp contact or elevated impedance levels (>5 kΩ) were excluded from further analysis. The overall rate of removal after ICA was less than 15%. Only the remaining epochs were part of subsequent signal processing and statistical analysis. Spectral power density (SPD) was computed using a classical estimation method derived from the Fourier Transform (FT), implemented in MATLAB (The MathWorks, Inc.). A classical estimator (i.e., parametric, Bartlett Periodogram, using non-overlapping 2 s long [480 samples] rectangular windows) was applied to the Power Spectral Density (PSD), estimated from the Fourier Transform (FT), which was performed using MATLAB (Mathworks, Inc.). Cortical modification analyses were performed for 1 s, 4 s, 7 s, and 9 s. For each epoch, the EEG recording began 2 s before the visual stimulus start (preparation for the task) and continued until the time 0 corresponding to the time estimation task execution [39,47].

The alpha frequency band (8–12 Hz) was analyzed at the F3 and F4 electrode sites, given its established association with neurobiological processes involved in temporal perception. The F3 and F4 electrode positions, corresponding to the DLPFC, were chosen for analysis due to their central involvement in key components of temporal processing—including working memory, executive control, attentional regulation, motor planning, and inhibitory mechanisms—particularly within the supra-second timescale [47,48,49].

### 2.8. Statistical Analysis

Genotypic distributions were assessed with Hardy–Weinberg equilibrium using the chi-square (χ^2^) test. The CV analysis was performed through descriptive measures (mean, median, standard deviation, and confidence interval) and the dispersion projection analysis for precision during the judgment of the time intervals by pseudo-sigma.

The data normality and homoscedasticity were previously ensured by the Shapiro–Wilk and Levene tests. Subsequently, we performed a Two-Way Mixed ANOVA with a group factor (10R-10R/6R-6R vs. no 10R-10R/6R-6R) and time factor: (1 s vs. 4 s vs. 7 s vs. 9 s). The EEG alpha band differences in each time interval were analyzed using Two-Way Mixed ANOVA with group factors: (10R-10R/6R-6R vs. no 10R-10R/6R-6R); and area factor: (right DLPFC vs. left DLPFC).

The interactions of two factors were investigated by univariate, followed by Tukey’s test for post hoc analysis, as well as Student’s Independent *t*-test, if necessary. The size effect was estimated by the partial square eta (ƞ^2^p) in the analysis of the variance and through Cohen’s d for the *t*-test. Statistical power and 95% CI were calculated for the dependent variables. Statistical power was interpreted as low power from 0.1 to 0.3; high power from 0.8 to 0.9. The magnitude of the effect for ƞ^2^p in the analysis of variances and Cohen’s d in t-tests were interpreted using the recommendations suggested by Cohen [50]: insignificant < 0.19; small from 0.20 to 0.49; the average from 0.50 to 0.79; large from 0.80 to 1.29. The 5% probability for type I error was adopted in all analyzes (*p* < 0.05), with alpha-Bonferroni correction for the interaction analysis, adjusting the *p*-value to *p* ≤ 0.0125.

Binary logistic regression models were analyzed to test an association between the study polymorphisms and the behavioral and neurophysiological variables. All analyzes were performed using SPSS for Windows version 20.0 (SPSS Inc., Chicago, IL, USA).

## 3. Results

### 3.1. Allele Frequency

Allele frequencies were performed by simple counting, based on functionality and frequency. The success rate for the genotyping of the *SLC6A3* gene polymorphisms was 100% (178/178 samples). However, 04 samples were excluded for 3′-UTR VNTR (accounting for 97.75% of analyzed samples) and none excluded for intron 8 VNTR. The observed allelic distribution indicated frequencies of 0.77 for the 10R allele and 0.23 for the 9R allele, whereas the 6R and 5R alleles exhibited frequencies of 0.82 and 0.18, respectively. All analyzed polymorphisms conformed to Hardy–Weinberg equilibrium within the study sample, as indicated by non-significant *p*-values (*p* > 0.05). (Table 1).

### 3.2. Time Estimation Variables

**Figure 5:**
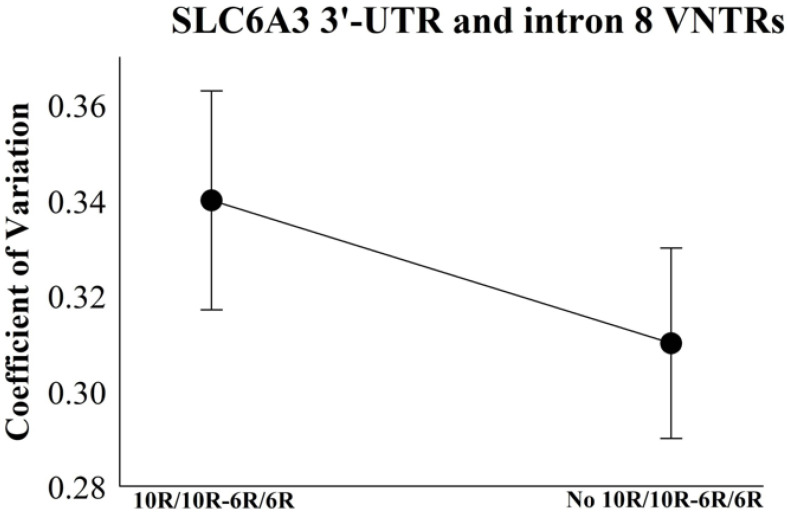
Coefficient of variation in relation to the time intervals for the 10R-10R/6R-6R and no 10R-10R/6R-6R groupings. The mean ± standard error represents the results.

The coefficient of variation was employed to quantify the degree of dispersion in temporal performance across genotype clusters defined by *SLC6A3* 3′-UTR and intron 8 VNTR polymorphisms, with less heterogeneity during the visual stimulus timing no 10R-10R/6R-6R cluster (Table 2 and Figure 5).

**Figure 6:**
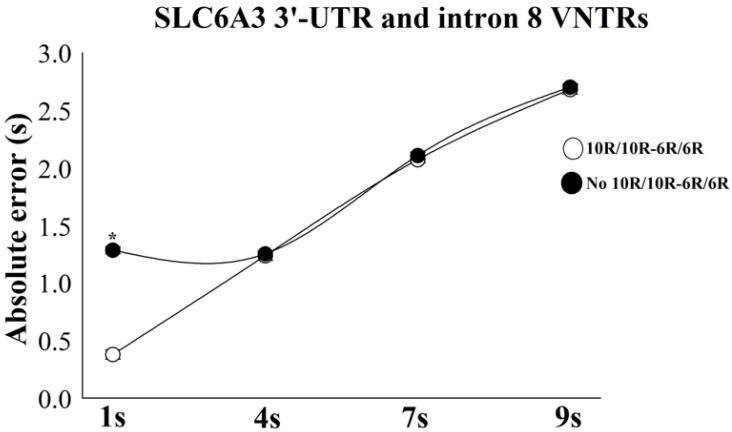
Time estimation performance between groups: 10R-10R/6R-6R (*n* = 71) vs. non-10R-10R/6R-6R (*n* = 103). Both groups increased the task’s errors as the time intervals increased. The results are expressed as mean ± standard error, with statistically significant difference for 1 s (*p* = 0.0002) indicated by an asterisk (*).

The analysis showed that the error increases as the target interval increases. In addition, a mixed two-way ANOVA for AE demonstrated an interaction between the combinatorial effect of *SLC6A3* 3′-UTR and intron 8 VNTRs vs. Time intervals, with [F(6) = 32.82; *p* = 0.002; ƞ^2^p = 0.51; power = 99.2%] (Figure 6). In the analysis of the interaction attributable to the combinatorial influence of *SLC6A3* 3′-UTR and intron 8 VNTR polymorphisms at each temporal condition using a between-subjects t-test, there was a significant result only in 1 s: [*t*(1) = 12.32; *p* = 0.0002; *d* = 0.56; 95% CI 0.39–1.32], with the cluster no 10R-10R/6R-6R obtaining an average error of 0.43 s higher than the group 10R-10R/6R-6R.

**Figure 7:**
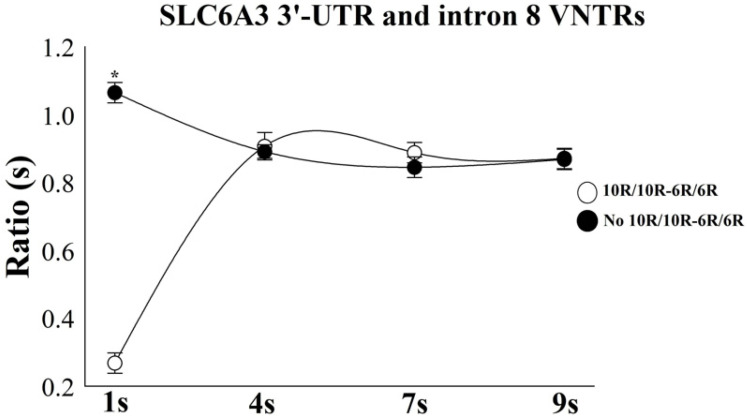
Time estimation pattern for groups: 10R-10R/6R-6R (*n* = 71) vs. non-10R-10R/6R-6R (*n* = 103). The participants grouped based on the combinatory effect 10R-10R/6R-6R, underestimated the interval of 1 s and overestimated the gaps of 4 s, 7 s, and 9 s. The results are expressed as mean ± standard error, with statistically significant difference for 1 s (*p* = 0.0001) indicated by an asterisk (*).

A two-way mixed ANOVA for the ratio demonstrated an interaction between the combinatorial effect of *SLC6A3* 3′-UTR and intron 8 VNTRs vs. Time intervals, with [F(6) = 19.48; *p* = 0.002; ƞ^2^p = 0.57; power = 98.3%] (Figure 7). An independent *t*-test was employed to evaluate the interaction effect of the *SLC6A3* polymorphism combination across individual time intervals, a significant result was found only in 1 s: [*t*(1) = 21.21; *p* = 0.0001; *d* = 0.57; 95% CI 0.34–0.72], with the cluster no 10R-10R/6R-6R demonstrating an overestimation in 0.79 s than the group 10R-10R/6R-6R.

The logistic regression model for the absolute error and ratio in relation to the combinatorial effect of the *SLC6A3* 3′-UTR and intron 8 VNTRs (cluster 10R-10R/6R-6R vs. cluster no 10R-10R/6R-6R) indicated an association probability only for 1 s in the AE (R_2_ = 0.56; B = 0.44; *p* = 0.003) and ratio (R_2_ = 0.62; B = 0.51; *p* = 0.0001) (Table 3).

### 3.3. EEG Alpha Power During Time Estimation

**Figure 8:**
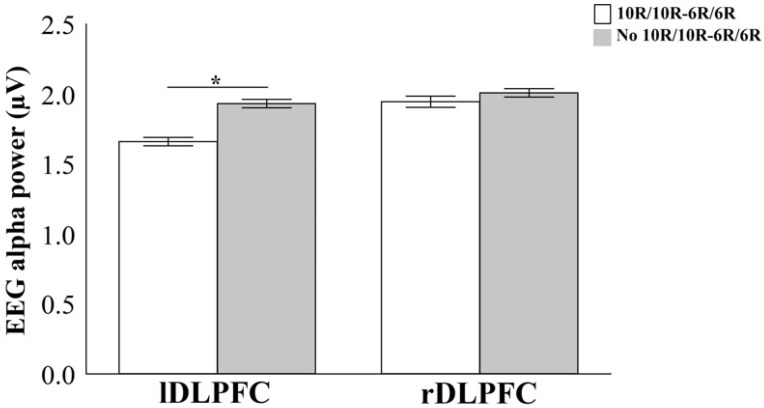
Representation of the EEG alpha band’s power dynamics in the DLPFC between the 10R-10R/6R-6R and no 10R-10R/6R-6R clusters. The results are expressed as mean ± standard error, with statistically significant differences (*p* = 0.0002) indicated by an asterisk (*). Note: lDLPFC: left Dorsolateral Prefrontal Cortex; rDLPFC: right Dorsolateral Prefrontal Cortex.

The results of the Two-Way Mixed ANOVA demonstrate interaction between the clustering factor *SLC6A3* 3′-UTR and intron 8 VNTRs and area factor only for the interval of 1 s: [F(5) = 18.54; *p* = 0.001; n^2^p = 0.53; power = 95.8%] (Figure 8). Independent *t*-test analysis of genotype differences within each region revealed a significant effect exclusively in the left DLPFC: [*t*(3) = 12.78; *p* = 0.0002; *d* = 0.57; 95% CI 0.31–0.52], with the cluster no 10R-10R/6R-6R obtaining an average power in alpha of 0.18µV higher than the group 10R-10R/6R-6R.

Logistic regression analysis for the combinatorial effect *SLC6A3* 3′-UTR and intron 8 VNTRs (cluster 10R-10R/6R-6R vs. no cluster 10R-10R/6R-6R) with changes in the alpha power of the EEG in the DLPFC in each target interval did not indicate a probability of association (*p* > 0.05) (Table 4).

## 4. Discussion

This study analyzed the relationship of EEG alpha-band potency in the DLPFC with the polymorphisms of the *SLC6A3* gene in healthy individuals during the time estimate’s interpretation.

### 4.1. Time Estimation Variables

A proportional relationship was observed between the duration of the time intervals and the magnitude of estimation errors, with longer intervals yielding greater inaccuracies. The AE and ratio metrics suggest that temporal estimation operates under an internal clock framework, modulated by dopaminergic signaling pathways, which function as a centralized mechanism linking stimulus processing to temporal judgment [51,52]. In the context of the internal clock model, the encoding of temporal information may arise from the accumulation of discrete pulses generated by a central pacemaker-like mechanism or on the synchronization of oscillatory patterns operating across various temporal scales—processes predominantly governed by neural oscillations and dopaminergic modulation in the prefrontal cortex [18]. Temporal processing appears to align with Scalar Expectancy Theory and beat-frequency models, indicating that pacemaker-driven timing and short-term encoding operate in synchrony with long-term memory retrieval mechanisms [53].

According to the striatal beat-frequency model of temporal estimation and dopaminergic modulation, timing arises from the detection of coincident oscillatory activity within cortico-striatal pathways. This framework posits that, upon initiation of a stimulus requiring temporal tracking, ensembles of cortical and thalamic neurons undergo phase resetting and initiate oscillations based on their intrinsic rhythmic properties. These oscillatory patterns tend to be more prominent in individuals carrying the 10R-10R/6R-6R genotype combination. At signal onset, dopamine release from the ventral tegmental area (VTA) is thought to contribute to the phase resetting of cortical neuronal oscillations, while simultaneously functioning as a ‘start signal’ for dopaminergic transmission by downregulating dopamine transporter (DAT) expression. At the onset of the stimulus, dopaminergic release from the substantia nigra pars compacta contributes to the reconfiguration of synaptic weights within the dorsal striatum, functioning as a modulatory signal that resets connectivity patterns critical for temporal processing [54,55]. This feature may explain the convergence between temporal interval processing and working memory, both of which appear to share overlapping neural coding mechanisms for stimulus duration. [56]. As a result, the spike-timing-based beat frequency framework aligns well with empirical findings across psychophysics, pharmacological studies, and anatomical models of temporal perception [57,58,59].

According to attention-level principles, fluctuations in attentional state modulate cortical activity by facilitating the accumulation of sensory information, the processing of external stimuli, and the execution of motor and cognitive tasks. These processes are influenced by neural excitability, the functioning of the default mode network, and variations associated with circadian rhythms [4,29,60]. The study exclusively included male participants to reduce potential sources of bias, given that prior research has demonstrated sex-related differences in neurophysiological function and dopaminergic neurochemistry across both human and animal models. All experimental sessions were scheduled at the same time of day to control for potential circadian influences. Strategies for minimizing bias and guiding data analysis followed a previously validated model applied in earlier studies with demonstrated effectiveness [10,41,52]. Accordingly, the findings suggest that the parameters derived from absolute error and ratio measures reflect stable temporal representation features within the supra-second time domain. Moreover, time intervals approaching 1 s tend to be synchronized automatically through neural integration mechanisms involving subcortical structures such as the basal ganglia and cerebellum. In intervals longer than 1 s, cognitive acuity decrease as the timing increases in the task, overestimating the time intervals [53,61].

Notably, dopaminergic modulation within cortico-striatal pathways has been identified as a key regulator of the internal clock’s pacing, particularly in temporal processing within the seconds-to-minutes range [62]. Thus, increases in dopamine’s ‘effective’ level increase clock speed and decrease temporal estimates’ uncertainty. In contrast, decreases in the ‘effective’ level of dopamine decrease clock speed and increases the uncertainty of temporal estimates [57,63].

Our findings extend previous evidence on dopaminergic modulation in the timing [16,17,18]. These results converge with neuroimaging and cognitive-genetic evidence, showing that the 9R allele is associated with increased DAT activity in healthy individuals, as revealed by Positron Emission Tomography data [19]. However, most of these studies investigated single genetic variants, whereas our results highlight that gene–gene interactions can differentially modulate supra-second interval processing. Thus, dopaminergic regulation of temporal cognition may involve interactive effects of multiple VNTRs within the DAT gene, complementing the state of the art.

### 4.2. EEG Alpha Power During Time Estimation

Regarding the association between neurophysiological and genetic variables, our findings observed different only left dorsolateral prefrontal cortex, with more significant activity for no 10R-10R/6R-6R. These results demonstrated that molecular changes that influence DAT-mediated neurotransmission in the left frontal cortex functions affect timing and coding in time estimation [64,65,66] since the frontal cortex is closely related to voluntary attention processes. The alpha frequency band is closely linked to the brain’s capacity to integrate and accumulate information during task-relevant processing [67].

The electrophysiological evidence in the present study indicates that left frontal EEG alpha power is closely linked to core cognitive processes essential for task execution. It has been proposed that the integrity of the cortico-striato-thalamo-cortical (CSTC) circuitry, particularly involving the left DLPFC, plays a critical role in temporal information synchronization, thereby contributing to perceptual capacity across different time scales. [68,69]. Transient fluctuations in absolute alpha power within the frontal cortex are regarded as objective neural markers of attentional engagement and perceptual processing of external stimuli. Thus, it is proposed that the distinction between cognitive self-regulation and responsiveness to prior environmental stimuli is mediated by a relative increase in neural activity within the left frontal cortex, owing to the information processing aimed at the time estimation (evidenced by differences between combinatorial effects of the *SLC6A3* gene). We suggest the left frontal EEG alpha power supports the encoding of temporal information at the supra-second scale, given its association with attentional processing of visual stimuli. This activity reflects a core neurobiological mechanism underlying internal clock oscillations during the execution of time estimation tasks [70,71].

We observed that alpha-band activity is modulated according to cognitive demand and the synchrony of visual stimulus perception, providing evidence for cortical activity suppression in areas not directly involved in task execution. Then, when the subject directs less attention to automatic tasks, the EEG alpha power decreases [36,71,72]. Our findings demonstrate the presence of regulator circuits, serving as cognitive resources for capturing and storing time intervals memory [73,74]. This result differs from some studies which establish a right hemisphere domain for temporal processing resources [75]. However, our findings explained the left frontal region is apt to encode information that will accumulate in the timing areas for sensory planning and integration of environmental stimuli [35,73].

Dopaminergic input to cortical regions originates predominantly from neurons located in the ventral tegmental area and the medial portion of the substantia nigra within the midbrain. In the prefrontal cortex, molecular changes in DAT modify CA2+-dependent dopaminergic signaling and uptake, blocked dopamine receptors impair temporal control of action, and attenuate temporal expectation during the interpretation of the target intervals [76]. Additionally, the no 10R-10R/6R-6R genotype group exhibits a reduction in ramping neuronal activity patterns, which are typically associated with the encoding of temporal information. The availability of dopamine expressed by the combinatorial effect of the no 10R-10R/6R-6R enhanced alpha power. The results suggest that prefrontal activity follows a U-shaped response to DAT signaling, with optimal dopamine levels being crucial for accurate temporal cognition [77].

Increased alpha power in the left DLPFC may indicate heightened dopaminergic activity. Based on neurobiology, we suggest that the left frontal cortex predominance may be associated with the D_1_-D_5_ dopaminergic receptors, in addition to high dopamine recycling activity mediated by DAT. Accordingly, our results suggest that both the magnitude and direction of EEG alpha power may reflect a behavioral phenotype related to time interval discrimination, by modulating dopaminergic pathways linking the frontal cortex to subcortical regions like the amygdala, basal ganglia, and cerebellum—thereby influencing information processing mechanisms involved in temporal decision-making [78,79,80].

### 4.3. Study Limitations

This study has some limitations. A key limitation is the homogeneity of the sample, restricted to young adult males. Age, sex, and hormonal factors are known to influence temporal processing through changes in neural oscillations and dopaminergic signaling [81,82,83]. Broader samples are needed to improve the generalizability of genetic findings on time perception. In addition, the non-association with sub-second level tasks, since it could provide a broader neurophysiology view in timing. Another limitation was the non-use of Electromyography (EMG) since the participants received the instruction to avoid nonspecific movements in the time perception task’s preparation and performance. However, the absence of electromyographic (EMG) recordings limited the confirmation of muscle activity during task execution. Additionally, the use of specific instruments to evaluate attentional and memory processes could have provided a more comprehensive understanding of the relationship between time estimation performance and dorsolateral prefrontal cortex activity. Finally, another limitation was to observe only the dorsolateral prefrontal cortex. However, this region was selected for its integrative role in temporal, cognitive, and sensorimotor processing.

## 5. Conclusions

The present study associates the molecular basis of the role in the dopamine transporter with the combinatory effect of *SLC6A3* 3′ UTR and intron 8 VNTRs polymorphism’s short-temporal scale judgment and EEG alpha power in the dorsolateral prefrontal cortex. The prefrontal cortex functions as a central integrative structure, processing sensory input and coordinating its transformation into goal-directed executive behaviors [79], proposed in the dopamine model’s transcriptional level, which modulate the neural chemistry in the circuits that involve the prefrontal cortex and basal ganglia action. It may indicate a continuous feedback loop in the frontal striatum-cortex pacemaker during the time-interval judgment [80].

The interpretation of prefrontal EEG power as an index of perceptual capacity remains limited by the current sample size and the scope of neurophysiological variables examined. Future studies should expand participant cohorts, incorporate a broader range of neural biomarkers associated with behavioral phenotypes, and employ time perception models capable of resolving sub-second intervals to enhance the precision and generalizability of these findings. Although few studies elucidate differences in the left dorsolateral prefrontal cortex via changes in the function of the association of polymorphisms of the *SLC6A3* gene, our findings support a neurophysiological basis of interval interpretation.

## Figures and Tables

**Table 1:** Counts and allele frequency.

Polymorphisms	Frequencies	Hardy–Weinberg Equilibrium
***SLC6A3* 3′-UTR VNTR**	(*n* = 174)	*p* = 0.230
10R/10R	93 (53.5%)	
10R/9R	64 (36.8%)	
9R/9R	17 (9.77%)	
**Alleles**		
10R	218 (76.7%)	
9R	66 (23.3%)	
***SLC6A3* intron 8 VNTR**	(*n* = 178)	*p* = 0.705
6R/6R	103 (57.8%)	
6R/5R	66 (37.1%)	
5R/5R	9 (5.1%)	
**Alleles**		
6R	239 (82.4%)	
5R	51 (17.6%)	

**Table 2:** Analysis of the variability of responses concerning the time interval for the combinatory effect of *SLC6A3* 3′-UTR and intron 8 VNTRs.

Coefficient of Variation—*SLC6A3* 3′-UTR and Intron 8 VNTRs						
Groups	Mean	SD	Median	Pseudo-Sigma	95% CI	
					Lower	High
**10R/10R-6R/6R**	0.34	0.25	0.25	0.23	0.31	0.34
**No 10R/10R-6R/6R**	0.31	0.19	0.26	0.18	0.30	0.33

Note: SD: Standard deviation; 95% CI: 95% confidence interval.

**Table 3:** Regression model for behavioral variables EA and Ratio based on the combinatorial effect for *SLC6A3* 3′-UTR and intron 8 VNTRs (analysis of cluster 10R-10R/6R-6R in relation to cluster no 10R-10R/6R-6R), *: *p* < 0.05.

Variables	*B*	S.E	Wald	*df*	*p*-Value	*Odds Ratio*	95%CI for *Odds Ratio*	
							Lower	High
AE 1 s	1.79	0.04	7.47	1	0.035 *	1.07	0.87	0.92
AE 4 s	0.74	0.03	2.13	1	0.144	0.94	1.01	1.15
AE 7 s	0.51	0.09	0.56	1	0.454	1.01	1.04	1.19
AE 9 s	0.35	0.01	0.72	1	0.394	0.98	1.11	1.22
*Ratio* 1 s	1.51	0.05	18.45	1	0.004 *	1.09	0.81	0.96
*Ratio* 7 s	−0.14	0.14	0.51	1	0.831	0.92	0.74	1.12
*Ratio* 9 s	−0.44	0.13	0.73	1	0.639	0.64	0.49	1.83
Constant	0.66	0.15	11.72	1	0.0001	0.87	-	-

Note: AE = Absolute error; B = Regression coefficient; S.E = Standard error; *df* = Degree of freedom. Significant differences (*p* < 0.05) are represented by the asterisk (*).

**Table 4:** Regression model for changes in the EEG’s alpha power based on the combinatorial effect for *SLC6A3* 3′-UTR and intron 8 VNTRs (cluster analysis 10R/10R-6R/6R about the cluster not 10R/10R-6R/6R).

Variables	*B*	S.E	Wald	*df*	*p*-Value	*Odds Ratio*	95%CI for *Odds Ratio*	
							Lower	Upper
lDLPFC 1 s	0.32	0.07	3.42	1	0.054	1.14	0.99	1.32
lDLPFC 4 s	0.16	0.04	0.62	1	0.806	1.01	0.89	1.15
lDLPFC 7 s	0.32	0.05	1.06	1	0.441	0.92	0.90	1.12
lDLPFC 9 s	0.71	0.04	0.73	1	0.145	0.98	1.11	1.22
rDLPFC 1 s	0.55	0.06	0.95	1	0.210	1.01	0.87	1.12
rDLPFC 4 s	0.40	0.05	1.02	1	0.439	0.99	0.72	1.14
rDLPFC 7 s	0.44	0.04	2.03	1	0.111	1.04	0.85	1.06
rDLPFC 9 s	0.80	0.03	0.72		0.543	0.96	0.88	1.11
Constant	0.62	0.05	1.98	1	0.042	1.07	-	-

Note: lDLPFC = left dorsolateral prefrontal cortex; rDLPFC = right dorsolateral prefrontal cortex; B = Regression coefficient; S.E = Standard error; *df* = Degree of freedom.

## Data Availability

Some methodological sections in this manuscript, particularly those involving EEG acquisition, genotyping protocols, and statistical analysis, replicate previously published technical descriptions from our research group. This reuse ensures consistency in complementary studies that share methodological frameworks. All reported data are original, and the results discussed are novel and specific to the present investigation.
